# Uptake of referrals for women with positive perinatal depression screening results and the effectiveness of interventions to increase uptake: a systematic review and meta-analysis

**DOI:** 10.1017/S2045796020000554

**Published:** 2020-07-17

**Authors:** WQ Xue, KK Cheng, D Xu, X Jin, WJ Gong

**Affiliations:** 1 Xiangya School of Public Health, Central South University, Changsha, China; 2 Institute of Applied Health Research, University of Birmingham, Birmingham, UK; 3 Sun Yat-sen Global Health Institute, School of Public Health and Institute of State Governance, Sun Yat-sen University, Guangzhou, China

**Keywords:** Depression, mental health, systematic reviews, women

## Abstract

**Aims:**

Perinatal depression threatens the health of maternal women and their offspring. Although screening programs for perinatal depression exist, non-uptake of referral to further mental health care after screening reduces the utility of these programs. Uptake rates among women with positive screening varied widely across studies and little is known about how to improve the uptake rate. This study aimed to systematically review the available evidence on uptake rates, estimate the pooled rate, identify interventions to improve uptake of referral and explore the effectiveness of those interventions.

**Methods:**

This systematic review has been registered in PROSPERO (registration number: CRD42019138095). We searched Pubmed, Web of Science, Cochrane Library, Ovid, Embase, CNKI, Wanfang Database and VIP Databases from database inception to January 13, 2019 and scanned reference lists of relevant researches for studies published in English or Chinese. Studies providing information on uptake rate and/or effectiveness of interventions on uptake of referral were eligible for inclusion. Studies were excluded if they did not report the details of the referral process or did not provide exact uptake rate. Data provided by observational studies and quasi-experimental studies were used to estimate the pooled uptake rate through meta-analysis. We also performed meta-regression and subgroup analyses to explore the potential source of heterogeneity. To evaluate the effectiveness of interventions, we conducted descriptive analyses instead of meta-analyses since there was only one randomised controlled trial (RCT).

**Results:**

Of 2302 records identified, 41 studies were eligible for inclusion, including 39 observational studies (*n* = 9337), one quasi-experimental study (*n* = 43) and one RCT (*n* = 555). All but two studies were conducted in high-income countries. The uptake rates reported by included studies varied widely and the pooled uptake rate of referral was 43% (95% confidence intervals [CI] 35–50%) by a random-effect model. Meta-regression and subgroup analyses both showed that referral to on-site assessment or treatment (60%, 95% CI 51–69%) had a significantly higher uptake rate than referral to mental health service (32%, 95% CI 23–41%) (odds ratio 1.31, 95% CI 1.13–1.52). The included RCT showed that the referral intervention significantly improved the uptake rate (*p* ＜ 0.01).

**Conclusions:**

Almost three-fifths of women with positive screening results do not take up the referral offers after perinatal depression screening. Referral to on-site assessment and treatment may improve uptake of referral, but the quality of evidence on interventions to increase uptake was weak. More robust studies are needed, especially in low-and middle-income countries.

## Introduction

Perinatal depression (PND) refers to depressive episodes that occur during pregnancy or after delivery. Affecting approximately 18% of all pregnancies worldwide (Gavin *et al*., 2005), this disorder is associated with many poor outcomes for women's maternal and physical health and the emotional and cognitive development of infants and children during their sensitive growth periods (Beardslee *et al*., 1998; Muzik and Borovska, 2010; Goodman *et al*., 2011). The early identification and timely intervention could improve the prognosis of PND means that in many countries screening for PND is incorporated as part of the routine perinatal care (Buist *et al*., 2008; Earls *et al*., 2010; Milgrom and Gemmill, 2014; O'Connor *et al*., 2016; Urato, 2017; ACOG, 2018). For screening to work, however, identification of women with positive screening results needs to be followed by timely intervention after referral. In this review, referral was defined as the process of recommending women to receive further mental health care after screening. The process of referral could be divided into three steps. First, the providers make the referral for women with positive screening results (step 1). Then, women accept the referral and try to access mental health services (step 2: ‘uptake’). Finally, relevant resources must be available to provide the necessary support (step 3). Steps 1 and 3 are both responsibilities of the healthcare system and routine screening should only be carried out if the prerequisites of these two are met. This review, therefore, focuses on step 2, namely uptake by women with positive screening results after being offered referrals. Low uptake rates reduce the overall effectiveness of screening. If a woman does not take up the offer of referral after the positive screening, the basis of introducing universal screening is weakened (Hewitt and Gilbody, 2009; Thombs *et al*., 2014).

Previous studies investigating the uptake of referrals after screening have reported that the uptake rates varied very widely (0% to 94%) (Tam *et al*., 2002; Miller *et al*., 2009). Uptake of referral after depression screening is a complex process. There are barriers related to patients and healthcare providers. These needed to be addressed in order to increase the uptake rate. A systematic review of uptake would help to inform the debate on the case for screening in those countries where screening is not yet routinely performed. In those where screening is already taking place, understanding the reasons behind low uptake rates and what interventions would increase these rates would be useful to improve the effectiveness of screening.

In this paper, we presented a systematic review that included studies reporting uptake rates after screening for PND and a meta-analysis on the overall uptake rate. We also explored the relationship between the type of referral interventions after administering the screening test and uptake. In addition, we summarised the reasons behind the lack of engagement with mental health care after referral. In contrast to related reviews (Byatt *et al*., 2015; Long *et al*., 2019), we have specifically focused on uptake of referral (step 2) for reasons stated above.

## Methods

### Literature review

This systematic review and meta-analysis was undertaken according to Preferred Reporting Items for Systematic Reviews and Meta-Analyses (PRISMA) guidelines. The protocol was registered in the PROSPERO database, number CRD42019138095. One of us who has experiences in women's mental health (WG) developed the search strategy and the full search strategy is available in Appendix A. In brief, we searched Pubmed, Web of Science, Cochrane Library, Ovid, Embase, CNKI, Wanfang Database and VIP Database for studies in English or Chinese from the inception of the database until January 13, 2019. In English database, we used the search terms (‘perinatal’, ‘pregnant’, ‘pregnancy’, ‘prenatal’, ‘antenatal’, ‘postnatal’ OR ‘postpartum’) AND (‘depression’ OR ‘depressive symptoms’) AND ‘screening’ AND (‘referral’, ‘referrals’, ‘refer’, ‘transfer’ OR ‘uptake’). We used the search terms (‘围产期’, ‘孕期’, ‘产前’ OR ‘产后’) AND (‘抑郁’ OR ‘抑郁症’) AND ‘筛查’ AND (‘转诊’ OR ‘转介’) in Chinese database.

### Inclusion and exclusion

After removing duplications, we reviewed each title and abstract based on inclusion and exclusion criteria. Inclusion criteria were: (1) language limited to English and Chinese; (2) participants were pregnant or postpartum (within 2 years of delivery) women who were screened positive for PND by any validated screening tool; (3) reported uptake rates (the number of women who accept the referral and try to access mental health service among women who were offered referral); (4) if there were interventions other than the administering of the screening test, the objective or one of the objectives of the interventions was to improve referral status. Exclusion criteria were: (1) no detail of referral process or exact data of uptake of referral were reported; (2) case report or case series. Then full-text articles were retrieved to determine eligibility criteria. Finally, references of retrieved full-text articles were screened for additional eligible publications. Investigators (WX and LL/QL/JW/PY/XM) independently assessed each study for inclusion. Disagreements were resolved through discussion with each other or consulting a third one (WG). When the full-text was not available (e.g. only the abstract was available), we would contact the author by email and if no reply was received within a month, the article would be excluded.

### Extraction

The following study-level characteristics were independently extracted (WX and LL/QL/JW/PY/XM) and disagreements were resolved via discussion or consulting a third one (WG): first author, publication year, study type, study country (countries are classified by income level according to World Bank Country and Lending Groups) (The World Bank Group, 2016), screening tool, sample size (number of women who were screened positive and were offered referral), time points of screening (prenatal, postnatal or perinatal), year of study, referral methods (referral to mental health services or on-site assessment or treatment), referral interventions, uptake of referral (number of women who accepted referrals and tried to access mental health service), referral uptake rates, the reasons for non-uptake and patient outcomes.

### Quality assessment

We used the Loney criteria to assess the quality of observational studies and quasi-experimental studies, and used Cochrane Risk of Bias Tool (ROS) to assess the quality of randomised controlled trials (RCTs) (Loney *et al*., 1998; Higgins *et al*., 2011). The Loney criteria included eight items on the risk of bias from three aspects: A. Validity of the study methods: (1) The study design and sampling method are appropriate for the research question. (2) The sampling frame is appropriate. (3) The sample size is adequate. (4) Objective, suitable and standard criteria are used for the measurement of the health outcome. (5) The health outcome is measured in an unbiased fashion. (6) The response rate is adequate and the refusers are described. B. Interpretation of the results: (7) The estimates of prevalence and incidence are given with confidence intervals and in detail by subgroup, if appropriate. C. Applicability of the findings: (8) The study subjects and the setting are described in detail. The maximal total score is eight points, with higher scores suggesting a lower risk of bias. ROS helps to evaluate the risk of bias from six aspects: selection bias, performance bias, detection bias, attrition bias, reporting bias and others. Each item was determined as ‘high risk of bias’, ‘low risk of bias’, ‘unclear risk of bias’. Assessment of bias was performed by two authors (WX and XJ) and disagreements were reconciled through discussion.

### Data analysis

In our review, prospective or retrospective studies that did not assign women to intervention or control groups at the referral stage were considered observational studies. Before and after comparison studies which examined the difference before and after the referral intervention took place were regarded as quasi-experimental studies. Studies that randomly allocated women to a referral intervention group or no intervention group, or to a high-intensive referral intervention group or low-intensive referral intervention group were categorised as RCT. The data provided by observational studies and quasi-experimental studies were used to estimate the pooled rate of uptake. RCT was used to evaluate the effect of interventions.

The ‘meta’ module in R-3.5.1 statistical software package was used for the calculation of the pooled rate of uptake. First, the uptake rates reported in each study were transformed using the Freeman-Tukey Double arcsine method according to the distribution of rates (Freeman and Turkey, 1950; Luo *et al*., 2013). Then we calculated the pooled rate and 95% confidence intervals (CIs) in a meta-analysis. Heterogeneity was assessed using Cochran's Q test, and quantified by the I^2^ value and tau^2^. If the heterogeneity results showed that *p* ⩽ 0.10 or *I*^2^ > 50% and suggested high heterogeneity, the random-effect model would be adopted. Otherwise, the fixed-effect model would be applied. Publication bias was evaluated by presenting a funnel plot and performing Egger's linear test. Sensitivity analysis was performed by serially removing studies one by one to explore the impact of doing so on the overall uptake rate. Any study for which removal substantially changed the uptake rate would be noted. Multivariate meta-regression was conducted to explore the source of heterogeneity and then the odds ratios (OR) were calculated through a formula: OR = exp(*β*) (*β*: regression coefficient). Through literature review, study country, time points of screening, referral methods and referral interventions were identified as predictor variables (Smith *et al*., 2009; Byatt *et al*., 2015; Gajaria and Ravindran, 2018; Savovic *et al*., 2018). Subgroup analyses were performed to estimate the pooled uptake rate with regard to the significant factors in meta-regression analyses and the differences between subgroups were investigated through chi-squared test (the significance level was *p* ＜ 0.05).

There were insufficient numbers of RCTs to allow for meta-analysis. Therefore, we conducted a descriptive analysis based on the uptake rates provided by the RCT in SPSS 18.0 to show the effectiveness of interventions.

## Results

### Search results

**Fig. 1.**
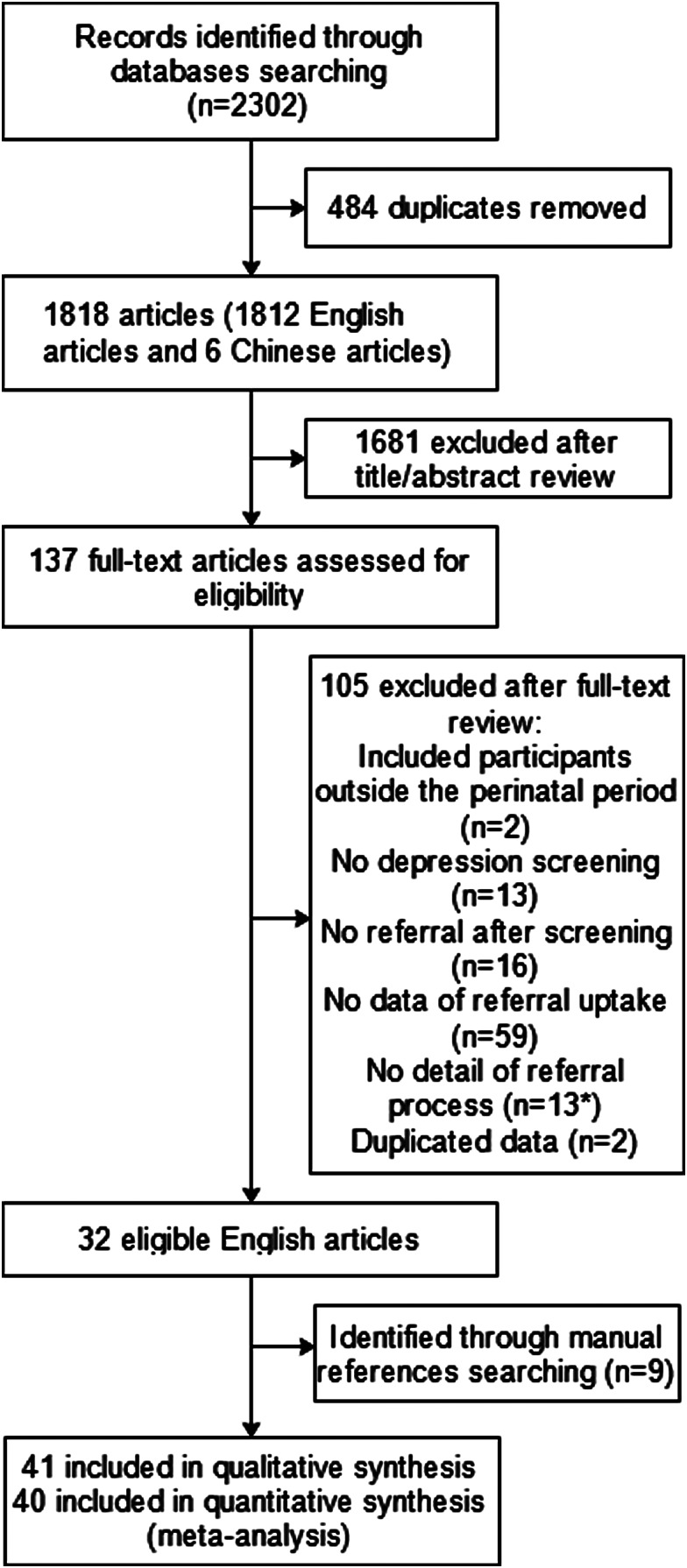
Article selection process. *Three articles were excluded because we did not get reply within 1 month after we contacted the authors by email.

The systematic literature search yielded 2302 articles, including 2296 English articles and six Chinese articles (Fig. 1). After removing duplicates, a total of 1818 references were identified. Of the 1818 reviewed, 1681 were eliminated after title/abstract review and 105 were eliminated after full-text reviews because they did not meet inclusion criteria (The main reasons for exclusion are shown in Fig. 1.) This resulted in 32 studies for inclusion. After additional searches, a further nine studies were included from reference lists of included papers. In total, 41 articles were included in this review, including one RCT, one before and after comparison study, and 39 observational studies.

### Quality assessment

Consensus was reached in over 90% among the two independent authors (WX and XJ) in quality assessment. In 40 observational or quasi-experimental studies, 12 of the 40 eligible studies scored five points, 15 scored four points, 11 scored three points and two scored two points based on the Loney criteria. For the validity of the study methods, 62% of studies (*n* = 25) used a biased sampling method, 95% of the studies (*n* = 38) did not apply an appropriate sampling frame, 72% (*n* = 29) had an insufficient sample size and 72% (*n* = 29) had low response rates or did not describe refusers. None provided confidence intervals. Three studies did not report the characteristics of study subjects. Result of the quality assessment of the RCT is shown in Appendix B. The RCT had ‘high risk’ in performance bias which meant it did not blind the randomisation status to participants or personnel.

### Study characteristics

Table 1 shows the characteristics of the eligible studies. Forty studies were observational or quasi-experimental studies with 9380 women who were screen positive. One was RCT with 555 women who were screen positive. Two-thirds of the 41 studies (*n* = 27) were carried out in the USA. The rest were conducted in Australia (*n* = 6), Iceland (*n* = 2) and there was one study each from Turkey, Singapore, New Zealand, China, Republic of South Africa and Israel. The sample size ranged from 5 to 1751 participants. Mean sample ages of women ranged from 23.4 to 35.7 years.

**Table 1.** Characteristics of studies included in this review

Author (year)	Study type, country and year of study	Screening tool and time points	Referral methods	Referral interventions	Sample size and uptake of referral	Uptake rate	Quality score
Lydsdottir *et al*. (2019)	Observational study; Iceland; 2006–2011	EPDS，DASS; 16 weeks gestation	Referral to mental health service	Provision of mental health consultation or diagnosis	396;273	69%	4
Kallem *et al*. (2019)	Observational study; USA; 2011–2014	EPDS; 2-month postnatal	Referral to mental health service	Provision of resources, assistance in referral	195;23	12%	4
Jarvis *et al*. (2018)	Observational study; USA; 2015–2016	EPDS; within 6 months postnatal	Referral to mental health service	Education, provision of resources, provision of mental health consultation or diagnosis, supportive treatment or referral to support group	37;6	16%	3
Bauer *et al*. (2017)	Observational study; USA; 2012–2014	EPDS; within 15 months postnatal	Referral to mental health service	Education, provision of resources	73;36	49%	3
Price *et al*. (2017)	Observational study; USA; /	BHRS; perinatal	Referral to mental health service	/	330;92	28%	5
Venkatesh *et al*. (2016)	Observational study; USA; 2010–2014	EPDS; 24–28 weeks gestation	On-site assessment or treatment	On-site assessment, assistance in referral	396;327	83%	5
		EPDS; 6-week postnatal			180;128	71%	
Mestad *et al*. (2016)	Observational study; USA; 2010	EPDS; first prenatal visit and at 26-week of pregnancy	On-site assessment or treatment	On-site assessment	55;35	64%	4
BenDavid *et al*. (2016)	Observational study; USA; 2013	EPDS; 2–3 weeks postnatal	Referral to mental health service	Education, provision of resources, assistance in referral, health care provider training	14;8	57%	5
Trost *et al*. (2016)	Observational study; USA; 2013–2014	EPDS; 2 weeks to 1 year postnatal	Referral to mental health service	Education, provision of resources, provision of mental health consultation or diagnosis, health care provider training	21;8	38%	3
Boyd *et al*. (2015)	Observational study; USA; /	EPDS; perinatal	Referral to mental health service	Education, assistance in referral	38;21	55%	3
Bina (2014)	Observational study; Israel; 2008–2009	EPDS; 6-week postnatal	Referral to mental health service	Education	88;21	24%	4
Emerson *et al*. (2014)	Observational study; USA; 2011–2012	EPDS; within 4 months postnatal	Referral to mental health service	Provision of resources, provision of mental health consultation or diagnosis	12;2	17%	3
Nelson *et al*. (2013)	Observational study; USA; 2008–2010	EPDS; first postnatal visit	Referral to mental health service	Assistance in referral	1106;250	23%	5
Stock *et al*. (2013)	Observational study; Australia; /	EPDS; 2 weeks to 6 months postnatal	On-site assessment or treatment	On-site assessment, provision of resources	39;27	69%	4
Wisner *et al*. (2013)	Observational study; USA; 2006–2010	EPDS; 4–6 weeks postnatal	On-site assessment or treatment	On-site assessment, education, health care provider training	1396;826	59%	4
Rowan *et al*. (2012)	Observational study; USA; 2008–2009	EPDS; first prenatal visit	Referral to mental health service	Assistance in referral	102;0	0%	4
		EPDS; 6-week postnatal			28;5	18%	
Segre *et al*. (2012)	Observational study; USA; 2002–2009	EPDS; prenatal to 2 years postnatal	On-site assessment or treatment	Provision of mental health consultation or diagnosis, supportive treatment or referral to support group, health care provider training, on-site assessment or treatment	573;271	47%	5
Honikman *et al*. (2012)	Observational study; South Africa; 2008–2011	EPDS; perinatal	On-site assessment or treatment	On-site assessment, health care provider training	1751;832	48%	5
Miller *et al*. (2012)	Before and after comparison; USA; 2008–2009	PHQ-9; postnatal	On-site assessment or treatment	On-site assessment (by behavioural health specialists)	10;1	10%	4
				On-site assessment (by perinatal care providers), health care provider training	33;28	85%	
Yawn *et al*. (2012)	Cluster RCTs; USA; 2006–2010	EPDS，PHQ-9; 5-12 weeks postnatal	On-site assessment or treatment	On-site assessment, health care provider training, provision of tools to facilitate the management of PND	322;194	60%	/
			Referral to mental health service	Short health care provider training	233;78	33%	
Milgrom *et al*. (2011)	Observational study; Australia; /	EPDS; 6 weeks-4 months postnatal	Referral to mental health service	Health care provider training, supportive treatment or referral to support group	333;68	20%	4
Burton *et al*. (2011)	Observational study; USA; 2006	EPDS; 36-week gestation	Referral to mental health service	Provision of mental health consultation or diagnosis	3;1	33%	5
		EPDS; 6 weeks postnatal			34;17	50%	
Chen *et al*. (2011)	Observational study; Singapore; 2008–2009	EPDS; 2 weeks to 6 months postnatal	On-site assessment or treatment	Education, supportive treatment or referral to support group, on-site assessment or treatment	126;41	33%	3
Reay *et al*. (2011)	Observational study; Australia; 2004	EPDS; prenatal and 6-8 weeks postnatal	Referral to mental health service	Provision of resources, assistance in referral	98;62	63%	4
Kim *et al*. (2010)	Observational study; USA; 2006–2007	EPDS; perinatal	Referral to mental health service	/	28;4	14%	3
Leung *et al*. (2011)	Observational study; Hong Kong, China; 2005–2006	EPDS; 2-month postnatal	On-site assessment or treatment	On-site assessment	67;51	76%	5
Horowitz *et al*. (2009)	Observational study; USA; 2004–2007	EPDS; 4-6 weeks postnatal	Referral to mental health service	Provision of mental health consultation or diagnosis, assistance in referral	674;185	27%	4
Miller *et al*. (2009)	Observational study; USA; /	EPDS; first prenatal visit, 28-week gestation and postnatal	On-site assessment or treatment	On-site assessment, health care provider training	174;164	94%	2
Orhon *et al*. (2007)	Observational study; Turkey; /	EPDS; within 1 year postnatal	Referral to mental health service	Provision of mental health consultation or diagnosis	35;28	80%	3
Harvey and Pun (2007)	Observational study; Australia; 2003–2005	EPDS; prenatal	Referral to mental health service	/	102;52	51%	4
Ingadottir and Thome (2006)	Observational study; Iceland; 2001–2002	EPDS; 9-week postnatal	On-site assessment or treatment	On-site assessment or treatment, supportive treatment or referral to support group	32;22	69%	5
Gordon *et al*. (2006)	Observational study; USA; 2003–2005	EPDS; 28-32 weeks of gestation, 6-week postnatal	Referral to mental health service	Provision of mental health consultation or diagnosis, assistance in referral, health care provider training	487;303	62%	5
Horowitz and Cousins (2006)	Observational study; USA; /	EPDS; 2-4 weeks postnatal	Referral to mental health service	Assistance in referral	117;21	18%	4
Flynn *et al*. (2006)	Observational study; USA; 2002–2004	EPDS; first prenatal visit	On-site assessment or treatment	Education, on-site assessment or treatment, assistance in referral	73;19	26%	4
Carter *et al*. (2005)	Observational study; New Zealand; /	EPDS; 12-22 weeks gestation	On-site assessment or treatment	On-site assessment	12;7	58%	3
Chaudron *et al*. (2004)	Observational study; USA; 1998–2001	EPDS; 1-year postnatal	On-site assessment or treatment	On-site assessment or treatment	16;7	44%	5
Miller *et al*. (2004)	Observational study; USA; 2003	EPDS; 4-6 weeks postnatal	Referral to mental health service	Education, provision of resources, assistance in referral	23;5	22%	5
Tam *et al*. (2002)	Observational study; USA; /	EPDS,BDI; 6-8 weeks, 4,6,9,12 months postnatal	Referral to mental health service	Provision of mental health consultation or diagnosis	5;0	0%	2
Fergerson *et al*. (2002)	Observational study; USA; 2001	EPDS; 4-6 weeks postnatal	On-site assessment or treatment	On-site assessment	11;5	45%	3
Rhonda *et al*. (1994)	Observational study; Australia; 1989	EPDS; 8-9 months postnatal	Referral to mental health service	/	45;6	13%	3
Robinson and Young (1982)	Observational study; Australia; 1980	SAD-G; 6-8 weeks postnatal	Referral to mental health service	Provision of mental health consultation or diagnosis	12;6	50%	4

Most studies (*n* = 38, 93%) used the Edinburgh Postnatal Depression Scale (EPDS) as the screening tool (Cox *et al*., 1987). The remaining used Patient Health Questionnaire (PHQ-9) (*n* = 2) (Wittkampf *et al*., 2007), Beck Depression Inventory (BDI) (*n* = 1) (Beck *et al*., 1961), Behavioural Health Risk Screen (BHRS) (*n* = 1) (Price *et al*., 2012), self-assessment of Depression, General Scale (SAD-G) (*n* = 1) (Snaith *et al*., 1976) and Depression Anxiety Stress Scales (DASS) (*n* = 1) (Lovibond and Lovibond, 1995). Time points of screening ranged from first antenatal checks to 2 years after delivery, which was broadly in line with the timing of prenatal/postnatal appointments or well-baby visits. Main screening sites were healthcare facilities, including obstetrics, paediatrics or neonatal intensive care units. After identifying women who were screened positive, programme members or health care providers who performed screening, including obstetricians, nurses, or midwives, would recommend referral. Some studies conducted remote screening through mail or telephone. The majority of studies referred women with positive screening results to mental health service for further mental health assessment or treatment. Providers of mental health services included mental health professionals (e.g. psychologists, psychiatrists), perinatal care providers (e.g. obstetricians, midwives), primary care providers (e.g. general practitioner) and behavioural health specialists. Some studies provided on-site assessment or treatment, which were mainly performed in the clinic by program members, social workers, or health care providers who conducted the screening. When women who were screened positive attended mental health department/clinic to undergo further assessment/treatment or received on-site assessment/treatment provided by studies, it constituted as uptake of referral in this review. In prospective studies, the outcomes of referral were mostly determined through follow-up by telephone. Of the prospective studies, 12 provided the length of follow-up which ranged from 2 weeks to 6 months. In retrospective studies, the outcomes of referral were always determined by reviewing the medical records.

Out of the 41 studies included in this review, 10 of them reported patient outcomes related to their depression (Appendix C). All ten studies showed improvement among patients who received further specialist support after referral.

### Pooled referral uptake rate

In 40 observational or quasi-experimental studies, three provided referral data of both prenatal and postnatal periods, one provided data of pre-intervention and post-intervention. Therefore, we had a total of 44 referral rates from 40 observational or quasi-experimental studies for the following analysis. The rates of referral uptake in different countries varied widely as Table 1 shows.

**Fig. 2.**
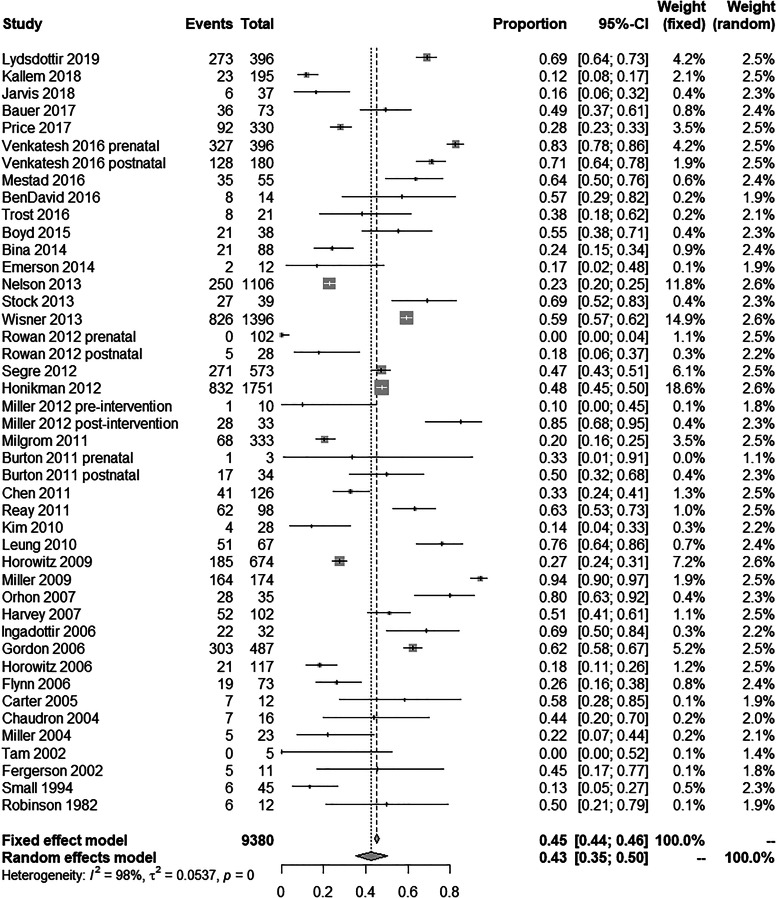
Forest plot of the results of the meta-analysis of referral uptake rates in included studies.

Figure 2 shows the uptake rates of all included observational or quasi-experimental studies and the pooled rate. Significant heterogeneity (*I*^2^ = 97.7%, *τ*^2^ = 0.0537, *p* ＜ 0.01) was observed across the included studies. The pooled uptake rate was 43% (95% CI 35–50%), using the random effect model.

### Publication bias and sensitivity analysis

The funnel plot was almost symmetrical suggesting that publication bias was likely to be small (see Appendix D). This was consistent with the result of the Egger's test (*t* = −0.32, *p* = 0.75).

Sensitivity analysis was performed through serially removing studies one by one. The I^2^ values ranged from 97.3% to 97.8%, the tau^2^ ranged from 0.0477 to 0.0655 and the combined referral rate ranged from 41% (95% CI 34–48%) to 44% (95% CI 37–52%), indicating that the results of the meta-analysis were reliable.

### Meta-regression analysis

The results of multivariate regression analysis are provided in Table 2. Referral methods were associated with the rate of uptake of referral but not with study country, time points of screening and referral interventions. The uptake rate where on-site assessment or treatment was used was significantly higher than the rate where the intervention was a referral to the mental health sector (OR = 1.31, 95% CI 1.13–1.52).

**Table 2.** Multivariate meta-regression analysis of referral rate

Variable	N	Regression coefficient (95% CI)	Statistical significance (*p*)
Study country^a^		Reference: Low- and Middle- income country −0.16 (−0.50, 0.19)	0.38
Low- and Middle- income country	2		
High-income country or region	42		
Time points of screening		Reference: Prenatal 0.04 (−0.08, 0.16)	0.53
Prenatal	8		
Postnatal	28		
Perinatal	8		
Referral methods		Reference: Referral to mental health service 0.27 (0.12, 0.42)	<0.01*
Referral to mental health service	27		
On-site assessment or treatment	17		
Number of referral interventions		Reference: No intervention ＜0.01 (−0.06, 0.08)	0.78
0	4		
1	17		
2	14		
3	5		
4	4		

a Income based on World Bank classifications.

*p < 0.05.

### Subgroup analyses

Subgroup analyses by referral methods showed significant differences in the uptake rate (*χ*^2^ = 17.95, *p* ＜ 0.01). Studies that provided on-site assessment or treatment had higher rates (60%, 95% CI 51–69%) than studies that referred women to mental health service (32%, 95% CI 23–41%).

### Referral interventions for women who were screened positive

Table 3 shows the referral interventions in eligible studies. Table 4 shows the characteristics of the RCT, which showed a significant difference in uptake rates between intervention and control group.

**Table 3.** The nature of referral interventions among included studies

Interventions	Definition
Education	Education on the definition, symptom and hazard of perinatal depression, prenatal depression or postnatal depression for women with positive screening results, mostly in the form of brochures (Miller *et al*., 2004; Flynn *et al*., 2006; Chen *et al*., 2011; Wisner *et al*., 2013; Bina, 2014; Boyd *et al*., 2015; BenDavid *et al*., 2016; Trost *et al*., 2016; Bauer *et al*., 2017; Jarvis *et al*., 2018).
On-site assessment or treatment	Mental health assessment or treatment at current clinic, mainly provided by perinatal care provider (eg, obstetrician or midwife), on-site social worker or researcher (Fergerson *et al*., 2002; Chaudron *et al*., 2004; Carter *et al*., 2005; Flynn *et al*., 2006; Ingadottir and Thome, 2006; Harvey and Pun, 2007; Horowitz *et al*., 2009; Miller *et al*., 2009; Chen *et al*., 2011; Leung *et al*., 2011; Honikman *et al*., 2012; Miller *et al*., 2012; Segre *et al*., 2012; Stock *et al*., 2013; Wisner *et al*., 2013; Mestad *et al*., 2016; Venkatesh *et al*., 2016).
Provision of resources	Providing mental health resources to women with positive screening results, mostly in the forms of a handout of PND resources (Miller *et al*., 2004; Reay *et al*., 2011; Stock *et al*., 2013; Emerson *et al*., 2014; BenDavid *et al*., 2016; Trost *et al*., 2016; Bauer *et al*., 2017; Jarvis *et al*., 2018; Kallem *et al*., 2019).
Health consultation or diagnosis	Mental health consultation or diagnosis for women with positive screening results provided by a mental health provider or social worker (Robinson and Young, 1982; Tam *et al*., 2002; Gordon *et al*., 2006; Orhon *et al*., 2007; Horowitz *et al*., 2009; Burton *et al*., 2011; Segre *et al*., 2012; Stock *et al*., 2013; Emerson *et al*., 2014; Trost *et al*., 2016; Jarvis *et al*., 2018; Lydsdottir *et al*., 2019).
Supportive treatment or referral to support group	Offering women with positive screening results in individual treatment (eg, psychotherapy or crisis intervention) or inviting women with positive screening results to participate in PND support group (Ingadottir and Thome, 2006; Chen *et al*., 2011; Milgrom *et al*., 2011; Segre *et al*., 2012; Jarvis *et al*., 2018).
Assistance in referral	Assistance by health care provider for mental health specialist appointment or further assessment or treatment (Miller *et al*., 2004; Flynn *et al*., 2006; Gordon *et al*., 2006; Horowitz and Cousins, 2006; Horowitz *et al*., 2009; Reay *et al*., 2011; Rowan *et al*., 2012; Nelson *et al*., 2013; Boyd *et al*., 2015; BenDavid *et al*., 2016; Venkatesh *et al*., 2016; Kallem *et al*., 2019).
Health care provider training	Training of health care provider in the knowledge and practice on screening, diagnosis, referral and management (Gordon *et al*., 2006; Miller *et al*., 2009; Milgrom *et al*., 2011; Honikman *et al*., 2012; Miller *et al*., 2012; Segre *et al*., 2012; Yawn *et al*., 2012; Wisner *et al*., 2013; BenDavid *et al*., 2016; Trost *et al*., 2016).

**Table 4.** Characteristics of the experimental study included

Author (year)	Study type, country and year of study	Screening tool and time points	Referral methods	Grouping	Referral interventions	Sample size, uptake of referral and uptake rate	*χ*^2^, *p*
Yawn *et al*. (2012)	Cluster RCTs; USA; 2006–2010	EPDS，PHQ-9; 5-12 weeks postnatal	Referral to mental health service	Control group	Short health care provider training	233;78;33%	38.77, <0.01*
			On-site assessment or treatment	Intervention group	On-site assessment, health care provider training, provision of tools to facilitate the management of PND	322;194;60%	

*p < 0.05.

### The reasons for non-uptake among screen-positive women

We examined the reasons for non-uptake at women or provider levels. For the former, the most frequent reasons were ‘lack of time’ and ‘perception that mood had improved’. Others cited ‘cost concerns’ or ‘transportation problems’. The perception of the nature of PND also affected referrals. Some declined referral because of stigma associated with psychiatric treatments. Some thought ‘it is normal to have some depression in the puerperium’ and therefore did not perceive the need for further health care. Women's preferences for the type of service offered also influenced the level of acceptance. It was reported that women were ‘not interested in receiving specialised services; home visitation was generally a much more acceptable referral’ and some women tended to ‘use their own resources’ instead of resorting to support provided by the research team. The reasons related to health care provider level mainly concerned the interaction between women with positive screening results and health care providers. Some women declined because of disagreement with their health care providers. For instance, some women wanted ‘a quick fix’ while psychiatrists ‘looked for something long term like counseling’. Meanwhile, the attitude of providers could influence referrals. ‘Women appreciated health professionals who gave their time, acknowledged their feelings and offered support’; and ‘when women's feelings were denied, when they felt unable or were not given the opportunity to talk, then the experience of seeking help could be a very negative one’.

## Discussion

### Principal findings

Our systematic review and meta-analysis identified 41 studies that reported the uptake rate of referral and the effect of interventions for women with positive PND screening results. These included 39 observational studies and one quasi-experimental study (total *N* = 9380 women with positive screening results) which were used to estimate the pooled uptake rate, and one RCT (*N* = 555 women with positive screening results) which was used to evaluate the effectiveness of referral interventions. The overall pooled uptake rate for women with positive screening results was 43% (95% CI 35–50%). Where the women were referred to was the most important determinant. Studies that provided on-site assessment or treatment had higher uptake rates than studies that referred the women to a separate mental health service. The RCT showed significant improvement in uptake rates after the implementation of referral interventions. The more frequently mentioned reasons for refusing referral were ‘lack of time’ and ‘perception that mood had improved’.

### Comparison with other studies and guidelines

Compared to Byatt *et al*. (2015) and Long *et al*. (2019), we focused on the uptake rate and the effectiveness of interventions. We showed that nearly 60% of women with positive screening results do not take up the offers for referrals to psychological/psychiatric services after screening. This uptake rate is considerably lower than those in other screening programs, such as cancer screening (Yabroff *et al*., 2003; Callen *et al*., 2012; Dalton, 2018). Such a low uptake rate will reduce the utility of screening programs. The low uptake raises the importance of finding the reasons for it and developing the strategy for improvement. We note that government or professional bodies in many high-income countries have recommended screening for PND (Appendix E). For seven of them, only five included any comments on the challenge presented by a low uptake rate or suggestions on how this can be improved. Our findings suggest that it is an important oversight that needs to be addressed.

Among the different methods of referral, on-site assessment or treatment appeared to be more effective. Previous studies have shown that women who received referrals to the same site as their prenatal or postnatal care were more likely than those referred offsite (Smith *et al*., 2009; Flynn *et al*., 2010; Price *et al*., 2017). Access to further assessments and treatment on-site is more convenient and would reduce the degree of stigma and therefore more acceptable to women. To achieve on-site referral, mental health services are needed in the perinatal health care settings. However, collocated mental health professionals are likely to be absent in almost all resource-poor areas (Patel and Prince, 2010). Equipping perinatal health providers with the capacity of providing basic mental health services may be a practical option. It is important to note that among the 40 eligible observational or quasi-experimental studies in our review, all but two were conducted in high-income countries or regions. As epidemiological evidence indicates PND is more common in low-and middle-income countries (Akhtar and Landeen, 2007; Shidhaye and Giri, 2014), and that many of these countries have huge populations (Patel and Prince, 2010), this inequity needs to be addressed urgently. An important global health priority would, therefore, be to conduct locally relevant research in low-and middle-income countries, especially the evaluation of the feasibility and cost-effectiveness of approaches to provide on-site assessment and treatment (for example, the use of mobile health technology or the training of perinatal care providers).

We found that the quality of the evidence on the effectiveness of the interventions to increase uptake was weak, as there was only one RCT that showed ‘high risk’ in performance bias. Furthermore, we note that interventions examined in previous studies tended to lack considerations for support from the woman's family, which was found to influence women's response to referral (Dennis, 2005; Ahmed *et al*., 2008). Another important consideration in improving the design of interventions is to collect qualitative information on the barriers to mental health services. In this review, among the two more frequent reasons mentioned were ‘lack of time’ and ‘perception that mood had improved’. The former may be addressed by improving the accessibility of health care services (including time, costs and transportation) (Lara-Cinisomo *et al*., 2014; Nagle and Farrelly, 2018; Jones, 2019). As the changes in circumstances for women and their families in the perinatal period can often be overwhelming, convenience when designing interventions would be important. The latter may reflect beliefs and cultural attitudes that may be modified by interventions aimed at improving knowledge about depression (Sword *et al*., 2008; Canty *et al*., 2019).

### Strengths and limitations

Strengths of this review included: first, our research question focused on the uptake of referral by women with positive PND screening results which has not been reported in previous reviews; second, we examined the effect of interventions and reasons of non-uptake to inform screening programs as well the design of further research to increase uptake of referral. Our review has several limitations. First, in the protocol registered with PROSPERO, the subject of the review is on referral in general. For reasons explained in detail in the Introduction, this review focuses on uptake rather than all three steps in the referral and treatment process. Second, the heterogeneity for the pooled uptake rate was high across the eligible studies. Only the referral method was identified as a moderator of the observed heterogeneity, suggesting that future studies should further explore the factors that contribute to the high heterogeneity. Third, the quality of the included studies was an important limitation in estimating the uptake of referral and assessing the effectiveness of interventions to increase uptake, with only one RCT in 41 eligible studies. Fourth, we searched for publications in English and Chinese only and those that would have missed articles in other languages. Finally, we only included information from published studies when assessing uptake rates. As we do not have information from the ongoing services worldwide, we cannot exclude the possibility that the uptake rates from these services may be higher than those reported in published studies.

### Implications

We find that almost three-fifths of women with positive perinatal depression screening results do not take up the offers for referrals to mental health service. In countries where screening is recommended, the reasons behind this low uptake should be assessed. Though efforts to address the challenge will be hampered by the weak overall quality of evidence on interventions to increase uptake, there is some suggestion that referral to on-site assessment and treatment may be helpful. Finally, as little is known in low-and middle-income countries where most affected women live, this issue should be addressed as an important global health research priority.

## Data

All the data supporting the findings of this meta-analysis have been provided in Tables and Figures.

## Appendix A: Search Strategy

Search Strategy (PubMed, -2019/01)

**Table:**

Step	Category	Terms
1	Terms for ‘perinatal’	(perinatal[all] OR pregnant[all] OR pregnancy[all] OR prenatal[all] OR antenatal[all] OR postnatal[all] OR postpartum[all])
2	Terms for ‘depression’	(depression[all] OR depressive symptoms[all])
3	Terms for ‘screening’	screening[all]
4	Terms for ‘referral’	(referral[all] OR referrals[all] OR refer[all] OR transfer[all] OR uptake[all])
5	Applies limits to combined ‘perinatal’ and ‘depression’ and ‘screening’ and ‘referral’	#1 AND #2 AND #3 AND #4

Search Strategy (Cochrane Library, -2019/01)

**Table:**

Step	Category	Terms
1	Terms for ‘perinatal’	(perinatal[ts] OR pregnant[ts] OR pregnancy[ts] OR prenatal[ts] OR antenatal[ts] OR postnatal[ts] OR postpartum[ts])
2	Terms for ‘depression’	(depression[ts] OR depressive symptoms[ts])
3	Terms for ‘screening’	screening[ts]
4	Terms for ‘referral’	(referral[ts] OR referrals[ts] OR refer[ts] OR transfer[ts] OR uptake[ts])
5	Applies limits to combined ‘perinatal’ and ‘depression’ and ‘screening’ and ‘referral’	#1 AND #2 AND #3 AND #4

Search Strategy (Web of Science, -2019/01)

**Table:**

Step	Category	Terms
1	Terms for ‘perinatal’	(perinatal[ts] OR pregnant[ts] OR pregnancy[ts] OR prenatal[ts] OR antenatal[ts] OR postnatal[ts] OR postpartum[ts])
2	Terms for ‘depression’	(depression[ts] OR depressive symptoms[ts])
3	Terms for ‘screening’	screening[ts]
4	Terms for ‘referral’	(referral[ts] OR referrals[ts] OR refer[ts] OR transfer[ts] OR uptake[ts])
5	Applies limits to combined ‘perinatal’ and ‘depression’ and ‘screening’ and ‘referral’	#1AND #2 AND #3 AND #4

Search Strategy (Embase, -2019/01)

**Table:**

Step	Category	Terms
1	Terms for ‘perinatal’	(perinatal[all] OR pregnant[all] OR pregnancy[all] OR prenatal[all] OR antenatal[all] OR postnatal[all] OR postpartum[all])
2	Terms for ‘depression’	(depression[all] OR depressive symptoms[all])
3	Terms for ‘screening’	screening[all]
4	Terms for ‘referral’	(referral[all] OR referrals[all] OR refer[all] OR transfer[all] OR uptake[all])
5	Applies limits to combined ‘perinatal’ and ‘depression’ and ‘screening’ and ‘referral’	#1 AND #2 AND #3 AND #4

Search Strategy (Ovid, -2019/01)

**Table:**

Step	Category	Terms
1	Terms for ‘perinatal’	(perinatal[mp] OR pregnant[mp] OR pregnancy[mp] OR prenatal[mp] OR antenatal[mp] OR postnatal[mp] OR postpartum[mp])
2	Terms for ‘depression’	(depression[mp] OR depressive symptoms[mp])
3	Terms for ‘screening’	screening[mp]
4	Terms for ‘referral’	(referral[mp] OR referrals[mp] OR refer[mp] OR transfer[mp] OR uptake[mp])
5	Applies limits to combined ‘perinatal’ and ‘depression’ and ‘screening’ and ‘referral’	#1 AND #2 AND #3 AND #4

Search Strategy (CNKI, -2019/01)

**Table:**

Step	Category	Terms
1	Applies limits to combined ‘围产期’ and ‘抑郁’ and ‘筛查’ and ‘转诊’	SU = ('围产期' + '孕期' + '产前' + '产后')*('抑郁' + '抑郁症')*'筛查'*('转诊' + '转介')

Search Strategy (Wanfang, -2019/01)

**Table:**

Step	Category	Terms
1	Applies limits to combined ‘围产期’ and ‘抑郁’ and ‘筛查’ and ‘转诊’	（‘围产期’ + ‘孕期’ + ‘产前’ + ‘产后’）*（‘抑郁’ + ‘抑郁症’）*‘筛查’*（‘转诊’ + ‘转介’）

Search Strategy (VIP, −2019/01)

**Table:**

Step	Category	Terms
1	Applies limits to combined ‘围产期’ and ‘抑郁’ and ‘筛查’ and ‘转诊’	U = (围产期 OR 孕期 OR 产前 OR 产后) AND U = (抑郁 OR 抑郁症) AND U = 筛查 AND U = (转诊 OR 转介)

## Appendix B: Results of quality assessment of the included RCT

**Table:**

	Selection bias		Performance bias	Detection bias	Attrition bias	Reporting bias	Other bias
	Random sequence generation	Allocation concealment					
Yawn *et al*. (2012)	Unclear risk	Unclear risk	High risk	Unclear risk	Unclear risk	Unclear risk	Low risk

## Appendix C: Patient outcomes reported by included studies

**Table:**

Author (Year)	Outcome domain	Outcome measure	Patient outcomes
Venkatesh *et al*. (2016)	Depression	EPDS	20% women who ‘screened positive antepartum and linked to mental health services’ experienced a reduction of scores to below the cut-off of 12.
Trost *et al*. (2016)	Depression	EPDS	‘Of 21 mothers initially EPDS1 who completed a follow-up call, 10 (48%) later screened negative.’
Boyd *et al*. (2015)	Depression	BDI	‘Depression scores decreased significantly from baseline to postintervention follow-up (*p* < 0.01)’
	Social support	Multidimensional Scale of Perceived Social Support	‘No change in social support over time.’
Segre *et al*. (2012)	Depression	EPDS	‘Treatment recipients experienced a decline in depressive symptoms between their first elevated EPDS score and their last available EPDS score (16.12 ± 3.62 *v*. 10.50 ± 5.73; *p* < 0.001).’
Yawn *et al*. (2012)	Depression	PHQ-9	‘Among the 654 women with elevated postpartum depression screening scores, those in the intervention practices had lower depressive symptom levels at 6 (*p* = 0.07) and 12 months’(*p* = 0.001) postpartum’
Milgrom *et al*. (2011)	Depression	BDI	‘This constituted a significant drop between baseline and post-study (mean reduction in BDI-II scores for all treatment groups combined = 17.3 points, 95% CI 14.2–20.5)’
	Anxiety and stress	DASS	There were ‘significant (p < 0.05) overall drop in anxiety over the course of the study.’
Chen *et al*. (2011)	Depression	EPDS	‘Of these 41 under clinical intervention, 78% (32) experiencing a reduction of scores to below the cut-off score of 13.’
	Function and symptoms	Global Assessment of Functioning Scale	‘76% (31) had a reduction in GAF scores.’
	Health status	EuroQol health index	‘68% (28) had a reduction in EQ-5D utility scores.’
Leung *et al*. (2011)	Depression	EPDS	At 18 months, there were 132 (80%) participants from the intervention and control groups with EPDS scores <10.
Orhon *et al*. (2007)	Depression	EPDS	‘In the overall sample, EPDS scores decreased on average by 7.4 ± 4.7 points.’
Ingadottir and Thome (2006)	Depression	EPDS	Fifteen weeks after birth, nine of 12 women at the experimental CHCs scored <12 on the EPDS but 1 of 10 scored <12 at control CHCs. Twenty-four weeks after birth, seven of eight women at experimental CHCs scored <12 on the EPDS but five of eight women scored <12 at control CHCs.

## Appendix E: Recommendations from different organisations on perinatal depression screening

**Table:**

Organisations	Condition	Is it recommended to screening	Suggestions for screening	Comments on the referral and uptake of referral	References
The American Academy of Pediatrics	Perinatal and postpartum depression	Screening recommended	‘The primary care pediatrician, by virtue of having a longitudinal relationship with families, has a unique opportunity to identify maternal depression and help prevent untoward developmental and mental health outcomes for the infant and family. Screening can be integrated into the well-child care schedule and included in the prenatal visit.’	‘ Intervention and referral are optimised by collaborative relationships with community resources and/or by co-located/integrated primary care and mental health practices.’	https://pediatrics.aappublications.org/content/126/5/1032
The Australian Clinical Practice Guidelines for Depression and Related Disorders in the Perinatal Period	Perinatal depression	Screening recommended	‘Consider routine, psychosocial assessment (EPDS and psychosocial questions as suggested in the Guidelines Appendix) for all women’ in the antenatal and postnatal period. ‘Timing of psychosocial assessment: early in pregnancy and 6–12 weeks after birth.’	‘Future research should include an examination of barriers to the uptake of referral.’	https://www.sciencedirect.com/science/article/pii/S1871519211002514
National Association of Pediatric Nurse Practitioners	Postnatal depression	Screening recommended	All pediatric nurse practitioners should be skilful ‘in screening mothers for risk of maternal depression’	None	https://www.sciencedirect.com/science/article/pii/S0891524510003159?via%3Dihub
UK National Institute for Health and Clinical Excellence	Perinatal depression	Screening recommended	Recommend identifying possible depression ‘at a woman's first contact with primary care, at her booking visit’ (first prenatal visit)’ and during the early postnatal period’ (usually at 4–6 wk and 3–4 mo)	‘Clinical networks should be established for perinatal mental health services, managed by a coordinating board of healthcare professionals, commissioners, managers, and service users and carers. These networks should provide clear referral and management protocols for services across all levels of the existing stepped-care frameworks for mental health problems, to ensure effective transfer of information and continuity of care.’	https://www.nice.org.uk/guidance/cg192
US Preventive Services Task Force	Depression in adults	Screening recommended	‘Recommend screening for depression in general adults population, including pregnant and postpartum women. Screening should be implemented with adequate systems in place to ensure accurate diagnosis, effective treatment and follow-up.’ ‘'Adequate systems in place' refers to having systems and clinical staff to ensure that patients are screened and, if they screen positive, are appropriately diagnosed and treated with evidence-based care or referred to a setting that can provide the necessary care.’	The trial assessing the effectiveness of screening and treatment in older adults ‘had a number of features that may affect its reliability, including external referrals for depression treatment, vert low uptake of treatment (19%), suggesting that the control and intervention group may have been different at baseline.’	10.1001/jama.2015.18392
Mental Health America	Perinatal depression	Screening recommended	‘Screening for perinatal mood and anxiety disorders and follow up care should be a required element as part of health home and general medical and mental health integration in all health plans.’	‘ Mental health professionals should be co-located within the settings where screening is performed to provide immediate evaluation, diagnosis, and treatment of mothers with positive screening results. This approach will reduce barriers to care, improve compliance, and provide the best outcomes for mothers and infants.’	https://www.mhanational.org/issues/position-statement-49-perinatal-mental-health
The American College of Obstetricians and Gynecologists	Perinatal depression	Screening recommended	It is ‘recommended that obstetrician–gynaecologists and other obstetric care providers screen patients at least once during the perinatal period for depression and anxiety symptoms using a standardised, validated tool.’	None	https://insights.ovid.com/crossref?an=00006250-201811000-00042
